# Comparison of Jaw Function and Mental Health Between Patients With Anterior Disc Displacement With and Without Reduction

**DOI:** 10.1002/cre2.70261

**Published:** 2025-12-15

**Authors:** Yuan Yao, Sha Sha Liu, Lei Jin, Ji Ling Ye, Zhong Yi Fang, Yang Yang, Li Li Xu, Bin Cai, Xin Jiang

**Affiliations:** ^1^ Department of Rehabilitation Medicine, Shanghai Ninth People's Hospital Shanghai Jiao Tong University School of Medicine Shanghai China

**Keywords:** anxiety, depression, jaw pain, mandibular function, somatic symptoms, temporomandibular disorders

## Abstract

**Objectives:**

Anterior disc displacement with reduction (ADDWR) and without reduction (ADDWOR) are two common clinical types of temporomandibular disorders. This study compared the levels of anxiety, depression, somatization, and jaw function of patients with ADDWR and ADDWOR.

**Material and Methods:**

From January 2023 to December 2024, 119 patients with ADDWR and 127 patients with ADDWOR were recruited from the Department of Rehabilitation Medicine at the Shanghai Ninth Peoples Hospital. All participants completed a single assessment, including a pain rating and several questionnaires to assess jaw dysfunction, depression, somatization, and anxiety levels. The scores and the grade distribution of somatization, depression, and anxiety of the two groups were compared.

**Results:**

Anxiety, depression, and somatization scores, as well as grade distributions, did not differ significantly between the ADDWR and ADDWOR groups. The scores for jaw function limitation, and pain on mouth opening and chewing were higher in the ADDWOR group than in the ADDWR group.

**Conclusions:**

Patients with ADDWOR demonstrated more severely impaired jaw function and greater pain intensity during mouth opening and mastication compared to the ADDWR group. However, no significant differences were observed between the groups in terms of anxiety, depression, or somatic symptoms.

## 1 Introduction

Temporomandibular disorder (TMD) is a group of diseases that involve the masticatory muscles and temporomandibular joint (TMJ) (Kapos et al. 2020; Schiffman et al. 2014). The prevalence rate of TMD ranges from 45% to 54% in different populations, and the peak age of onset is between 20 and 30 years of age, with a higher incidence in women than in men (Shao et al. 2023; Talaat et al. 2018). Anterior disc displacement with reduction (ADDWR) and anterior disc displacement without reduction (ADDWOR) are the two most common clinical types of intra‐articular disorders of the TMJ, accounting for approximately 50% of TMD (Wieckiewicz et al. 2014; Miyake et al. 2004).

Patients with ADDWR typically present with joint clicking during mouth opening, closing, or mastication, sometimes accompanied by pain (Mohamed et al. 2022; Poluha et al. 2019). Owing to these symptoms, patients may develop apprehension about opening their mouth wide or chewing food, consequently leading to impaired jaw function (Kalaykova et al. 2011; Marpaung et al. 2018). Patients with ADDWOR experience clinically significant mouth opening limitation accompanied by pain during both jaw movement and mastication, which collectively result in substantial restrictions in function (Miernik and Więckiewicz 2015; He et al. 2022) and affect the quality of life (Bitiniene et al. 2018). In recent years, the number of patients presenting with TMJ clicking has gradually increased. According to conventional wisdom the functional level of patients with ADDWR is better than that of patients with ADDWOR (Topaloglu Yasan et al. 2023). However, few studies have compared the functional levels of patients with ADDWR and those with ADDWOR.

Many patients with TMD experience psychological comorbidities such as anxiety, depression, and somatization in addition to functional jaw impairment (Salinas Fredricson et al. 2022). These psychological problems play an important role in the onset of TMD and symptom persistence (Fillingim et al. 2013). Negative emotions such as stress can potentially alter the pain perception threshold of the central nervous system, increase the intensity of parafunctional habits as well as the fatigue and tightness of the masticatory muscles (Monteiro et al. 2011). Consequently, mental health assessment is an essential component in TMD management. However, despite ADDWR and ADDWOR being the two most common clinical subtypes of TMD, few published studies have compared the mental health of these two subgroups.

Therefore, the purpose of this study was to compare the level of jaw function and mental health of patients with ADDWR with those of patients with ADDWOR. The hypothesis was that jaw function as well as mental health would be worse in patients with ADDWOR than in those with ADDWR.

## 2 Materials and Methods

### 2.1 Participants

This study was a cross‐sectional observational study. The study was approved by the Medical Ethics Committee of the Ninth People's Hospital Affiliated to Shanghai Jiao Tong University School of Medicine (approval number: SH9H‐2019‐T316‐4). All patients were informed of the overall purpose and procedures of the study and provided voluntary written informed consent.

From January 2023 to December 2024, patients with ADDWR and ADDWOR were recruited from the Department of Rehabilitation Medicine at the Shanghai Ninth Peoples Hospital. Patients aged between 18 and 45 years were considered for inclusion if they were diagnosed with ADDWR or ADDWOR. All diagnoses were required to be confirmed by magnetic resonance imaging. Patients with rheumatoid arthritis; vital organ impairments, including injury of the liver, spleen, bladder, or lung; nervous system problems, including cerebrovascular disease, periodic paralysis, progressive muscular dystrophy, and dystaxia; those who were pregnant; and those with limited cognitive ability were excluded.

### 2.2 Procedures

Data were collected on participants’ age, other demographic information, height, and weight. They were asked to report their pain scores and complete several questionnaires, as described below. The investigation was conducted in the Department of Rehabilitation Medicine at the Shanghai Ninth Peoples Hospital.

### 2.3 Measures

Participants reported the severity of their pain using a visual analog scale (VAS). The visual pain ratings increased horizontally from 0 to 10 (left to right), with 0 representing no pain and 10 representing severe pain. Participants selected the rating that corresponded to the pain intensity of their TMJ at rest, at maximal mouth opening, and during chewing. We repeated the assessment three times and calculated the mean value.

The patients completed the following self‐reported measures: the jaw functional limitation scale (JFLS), patient health questionnaire‐15 (PHQ‐15), patient health questionnaire‐9 (PHQ‐9), and generalized anxiety disorder‐7 (GAD‐7).

The JFLS comprised 20 items that evaluated chewing functions, eating with the mouth open, speaking, and other activities. Each item was scored on a scale of 0–10 points, with higher scores indicating more restricted function. All items were included to show the patient's overall mandibular function (Xu et al. 2019).

The PHQ‐15 was used to measure somatic symptoms during the past month. The PHQ‐15 comprised 15 items, each rated from 0 to 2 points, for a total of 30 points, with higher scores indicating more severe somatic symptoms. The total scores were graded as no somatization (0–4 points), mild somatization (5–9 points), for moderate somatization (10–14 points), or severe somatization (15–30 points) (Kroenke et al. 2002).

The PHQ‐9 was used to assess symptoms of depression in patients during the past 2 weeks. Nine items were each rated from 0 to 3 points, for a total of 27 points, with higher scores indicating more severe depression. The total scores were graded as no depression (0–4 points), mild depression (5–9 points), moderate depression (10–14 points), moderate to severe depression (15–19 points), or severe depression (20–27 points) (Kroenke et al. 2001).

The GAD‐7 questionnaire was used to assess symptoms of anxiety in patients during the previous 2 weeks. Seven items were each rated from 0 to 3 points, for a total of 21 points, with higher scores indicating a higher level of anxiety. The total scores were graded as no anxiety (0–4 points), mild anxiety (5–9 points), moderate anxiety (10–14 points), or severe anxiety (15–21 points) (Toussaint et al. 2020).

### 2.4 Statistical Analysis

The PASS Statistics package version 15.0 (NCSS Statistical Software, Kaysville, UT, USA) was used for sample size calculation. The data were analyzed using IBM SPSS Statistics package version 20.0 (IBM Corp., Armonk, NY, USA). Continuous data were reported as the mean and standard deviation or median (interquartile range) based on the normality, which was examined using the Kolmogorov–Smirnov test. According to the data distribution characteristics, the score differences between the two groups for each measure were tested using non‐parametric tests or independent sample *t*‐tests. Differences between the two groups in terms of gender and the grade distributions of somatization, depression, and anxiety were analyzed using the chi‐square test. Statistical significance was set at *p* < 0.05.

The results were reported according to the STROBE guidelines.

## 3 Results

A total of 122 patients with ADDWR were recruited, of whom 3 with incomplete questionnaires were excluded, leaving 119 patients with ADDWR in the analysis. A total of 135 patients with ADDWOR were recruited, of whom 5 with incomplete questionnaires and 3 patients with unstable symptoms were excluded, leaving 127 patients with ADDWOR in the analysis. The characteristics of the two groups of patients are shown in Table 1. Age, sex, and BMI did not differ significantly between the two groups. While the ADDWOR group reported significantly greater VAS at maximal mouth opening and JFLS scores (both *p* < 0.001), measures including VAS at rest, VAS during chewing, and the scores as well as grade distributions of the PHQ‐15, PHQ‐9, and GAD‐7 all showed no significant differences between the groups (Tables 2 and 3).

**Table 1:** Comparison of characteristics of patients with ADDWR and ADDWOR.

Characteristic	ADDWR (*N* = 119)	ADDWOR (*N* = 127)	*Z*/*χ* ^2^	*p* value
Gender			0.039	0.844
Male	16	16		
Female	103	111		
Age (years)	28.82 ± 9.38	28.87 ± 8.60	−0.530	0.596
BMI (kg/m^2^)	20.69 ± 4.62	20.57 ± 4.31	−1.212	0.225

Abbreviations: ADDWOR, anterior disc displacement without reduction; ADDWR, anterior disc displacement with reduction; BMI, body mass index.

**Table 2:** Comparison of the VAS, JFLS, PHQ‐15, PHQ‐9, and GAD‐7 scores of patients with ADDWR and ADDWOR.

	ADDWR	ADDWOR	*Z* value	*p* value
VAS				
Rest	0.40 ± 1.00	0.46 ± 1.06	−0.580	0.562
MMO	1.08 ± 1.53	2.32 ± 1.94	−5.247	< 0.001*
Chewing	1.26 ± 1.64	2.00 ± 2.10	−2.660	0.008*
JFLS	29.43 ± 24.25	47.50 ± 26.78	−5.079	< 0.001*
PHQ‐15	6.31 ± 7.14	5.96 ± 4.87	−0.397	0.692
PHQ‐9	5.28 ± 6.62	5.37 ± 4.73	−1.083	0.279
GAD‐7	3.68 ± 4.13	3.79 ± 3.91	−0.532	0.595

Abbreviations: ADDWOR, anterior disc displacement without reduction; ADDWR, anterior disc displacement with reduction; GAD, generalized anxiety disorder; JFLS, jaw functional limitation scale; MMO, maximal mouth opening; PHQ, patient health questionnaire; VAS, visual analog scale.

* The difference between the groups was statistically significant (*p* < 0.05).

**Table 3:** Score distributions and comparisons between patients with ADDWR and ADDWOR using chi‐square tests.

	ADDWR	ADDWOR	*χ* ^2^	*p* value
PHQ‐15			0.004	0.950
Normal	52	56		
Symptomatic	67	71		
Mild	47	52		
Moderate	12	13		
Severe	8	6		
PHQ‐9			0.465	0.495
Normal	67	66		
Symptomatic	52	61		
Mild	37	46		
Moderate	11	9		
Moderate to severe	1	4		
Severe	3	2		
GAD‐7			0.006	0.938
Normal	83	88		
Symptomatic	36	39		
Mild	26	28		
Moderate	7	8		
Severe	3	3		

Abbreviations: ADDWOR, anterior disc displacement without reduction; ADDWR, anterior disc displacement with reduction; GAD, generalized anxiety disorder; PHQ, patient health questionnaire.

## 4 Discussion

This study revealed that the level of jaw function in the ADDWOR group was lower than that in the ADDWR group, as reflected by higher JFLS and pain at the maximal mouth opening and chewing VAS scores in the ADDWOR group than in the ADDWR group, but the levels of anxiety, depression, and somatization did not differ significantly between the two groups.

Pain in the masticatory muscles or TMJ is a common complaint among patients with TMD (Yap et al. 2024). The pain scores at maximal mouth opening and chewing was significantly higher in patients with ADDWOR than in those with ADDWR. Due to anterior disc displacement, the retrodiscal tissue is passively stretched during mouth opening and subjected to pressure during chewing in patients with ADDWOR, which can lead to pain (Pérez del Palomar and Doblaré 2007). This explains why pain at the end of mouth opening and during chewing was generally higher in patients with ADDWOR than in those with ADDWR. However, the mean scores differed by less than 2 points between the two groups, indicating that this difference was not clinically significant (Dworkin et al. 2008). Notably, patients with ADDWR generally have no limitation in mouth opening, and experience no pain or only mild pain at the end of mouth opening or during chewing. Therefore, the pain level is generally believed to be lower in patients with ADDWR than in patients with ADDWOR. However, in this study, the mean pain scores of patients with ADDWOR were not significantly higher than those of patients with ADDWR. This may be because the patients with ADDWR in this study were individuals seeking medical treatment, who, in addition to clicking sounds, were experiencing a certain amount of pain, and thus had more severe symptoms that that of individuals with ADDWR in the general population. The resting pain scores in both groups were lower than the pain scores during mouth opening and chewing with no significant differences between groups, consistent with the general symptom characteristics of TMD.

The mean JFLS score was higher in patients with ADDWOR than in patients with ADDWR, indicating that the level of jaw function was significantly lower in patients with ADDWOR than in patients with ADDWR. Patients with ADDWOR experience limited mouth opening and notable pain at the end of mouth opening, which restricts activities requiring wide mouth opening, such as opening the mouth wide to eat a sandwich or yawning (Yap et al. 2024; Kliangkaeo et al. 2024). In contrast, patients with ADDWR have less restriction in these behaviors. For this reason, the treatment for patients with ADDWR mainly involves general measures to maintain stability, such as oral behavior education, modality therapy and exercise training, without excessive intervention (Ekici et al. 2022; Olbort et al. 2023; Wänman and Marklund 2020). Helping patients transition from the ADDWOR to the ADDWR state may be a goal of treatment. However, further research is required to assess whether patients who transition from the ADDWOR state to the ADDWR state can maintain the ADDWR state.

This study also compared the anxiety, depression, and somatization status of the two patient groups. The depression and anxiety levels did not differ significantly between the two groups, contrary to our hypothesis that patients with ADDWOR might experience more prominent negative emotions such as anxiety and depression than those with ADDWR owing to their more severe functional limitations. The overall scores of the psychological questionnaires for both groups fell within the mild range. Previous studies have suggested that patients with TMD have a high incidence of psychological comorbidities, (Salinas Fredricson et al. 2022; Yeung et al. 2017) but few studies have compared the depression and anxiety levels of patients with ADDWR with those of patients with ADDWOR. Most patients with chronic pain have some psychological issues (Macfarlane et al. 2004; LeResche et al. 2007). The results of this study indicate that the psychological needs of patients with ADDWR and ADDWOR are similar.

This study has some limitations. It only compared the depression and anxiety levels of patients with ADDWR with those of patients with ADDWOR and did not compare these levels with those of healthy individuals without TMD. Additionally, many factors including symptom duration influence jaw function and depression and anxiety levels, and we did not stratify the analysis by potential effect modifiers in this study. Future studies are required to address these limitations.

## 5 Conclusion

This study compared the jaw function, pain, somatization, depression, and anxiety levels of patients with ADDWR with those of patients with ADDWOR. The overall level of jaw function was lower in patients with ADDWOR than in those with ADDWR, with higher pain scores during maximal mouth opening and chewing and lower jaw function scores in patients with ADDWOR. However, the depression, anxiety somatization levels did not differ significantly between the two groups.

## Author Contributions

Yuan Yao contributed to conception, design, data acquisition and interpretation, performed all statistical analyses, and drafted and critically revised the manuscript. Jin Lei and Sha Sha Liu contributed to conception, design, data acquisition, and interpretation. Ji Ling Ye and Zhong Yi Fang contributed to design, performed all statistical analyses, and drafted and critically revised the manuscript. Yang Yang and Li Li Xu contributed to conception, data acquisition, and interpretation. Bin Cai contributed to conception, design, data acquisition, and interpretation. Xin Jiang contributed to conception, design, and critically revised the manuscript. All authors read and approved the final version of the manuscript.

## Conflicts of Interest

The authors declare no conflicts of interest.

## Data Availability

The data that support the findings of this study are available from the corresponding author upon reasonable request.
